# Tivozanib Monotherapy in the Frontline Setting for Patients with Metastatic Renal Cell Carcinoma and Favorable Prognosis

**DOI:** 10.1007/s11912-024-01613-7

**Published:** 2024-11-20

**Authors:** Ricky Frazer, José Ángel Arranz, Sergio Vázquez Estévez, Omi Parikh, Laura-Maria Krabbe, Naveen S. Vasudev, Christian Doehn, Norbert Marschner, Tom Waddell, Will Ince, Peter J. Goebell

**Affiliations:** 1 Velindre Cancer Centre, Cardiff, CF14 2TL UK; 2 Hospital General Universitario Gregorio Marañón, Madrid, Spain; 3 Hospital Universitario Lucus Augusti (HULA), Lugo, Spain; 4 Lancashire Teaching Hospitals NHS Foundation Trust, Chorley, UK; 5 Klinik für Urologie, Vivantes Humboldt-Klinikum, Berlin, Germany; 6 Leeds Institute of Medical Research at St James’s, Leeds, UK; 7 Urologikum Lübeck, Lübeck, Germany; 8 Clinical Research Institute IOMEDICO, Freiburg, Germany; 9 The Christie NHS Foundation Trust, Manchester, UK; 10 Department of Oncology, Cambridge University NHS Foundation Trust, Cambridge, UK; 11 Uniklinik Erlangen, Urologische und Kinderurologische Klinik, Erlangen, Germany

**Keywords:** Metastatic renal cell carcinoma, First-line treatment, Tyrosine kinase inhibitors, Checkpoint inhibitors, Favorable risk, Tivozanib

## Abstract

**Purpose of Review:**

In this review, we discuss which patients with metastatic clear cell renal cell carcinoma (mRCC) may be most suitable for frontline tyrosine kinase inhibitor (TKI) monotherapy, a treatment option supported by emerging long-term efficacy data including overall survival and quality of life. We specifically focus on tivozanib, a potent and selective inhibitor of vascular endothelial growth factor receptor, which has comparable efficacy to other single-agent TKIs in frontline treatment for mRCC while exhibiting fewer off-target side effects.

**Recent Findings:**

Combination therapy with TKIs and checkpoint inhibitors (CPIs) and CPI/CPI combination therapies, as well as TKI monotherapy are recommended frontline treatment options for mRCC. Treatment decisions are complex and based on several factors, including the patient’s International Metastatic RCC Database Consortium risk status, age, comorbidities, and personal preferences related to response, tolerability, and quality of life. TKIs not only serve as backbone of most combination therapies for mRCC, but also remain a viable monotherapy option in the first-line setting for patients in favorable risk groups and those with contraindications to CPI combination therapies.

**Summary:**

Given that overall survival benefits have not yet been confirmed for CPI-containing combination regimens in favorable risk patients, we argue that frontline single-agent TKI treatment remains a standard of care option for these patients. This is supported by treatment guidelines, even in the era of TKI/CPI combination therapies.

## Introduction

Treatment guidelines for metastatic renal cell carcinoma (mRCC) recommend several treatment options, including tyrosine kinase inhibitors (TKIs) and/or checkpoint inhibitors (CPIs) [1–5]. Factors affecting treatment choice include International Metastatic RCC Database Consortium (IMDC) risk status, potential side effect profile, predicted response, patient choice, survival outcomes, and others [6]. Combination therapy using CPI with or without TKIs have demonstrated superiority over sunitinib as first-line treatment for patients with mRCC [6]; however, TKI monotherapy remains an effective and valid treatment option for selected patients [7].

The objective of this paper is to review the published literature for TKI monotherapy, with a focus on tivozanib, in the context of other systemic anti-cancer therapies for first-line treatment of mRCC and in the light of emerging evidence on their long-term use and subsequent outcomes.

## The mRCC Treatment Landscape

**Fig. 1:**
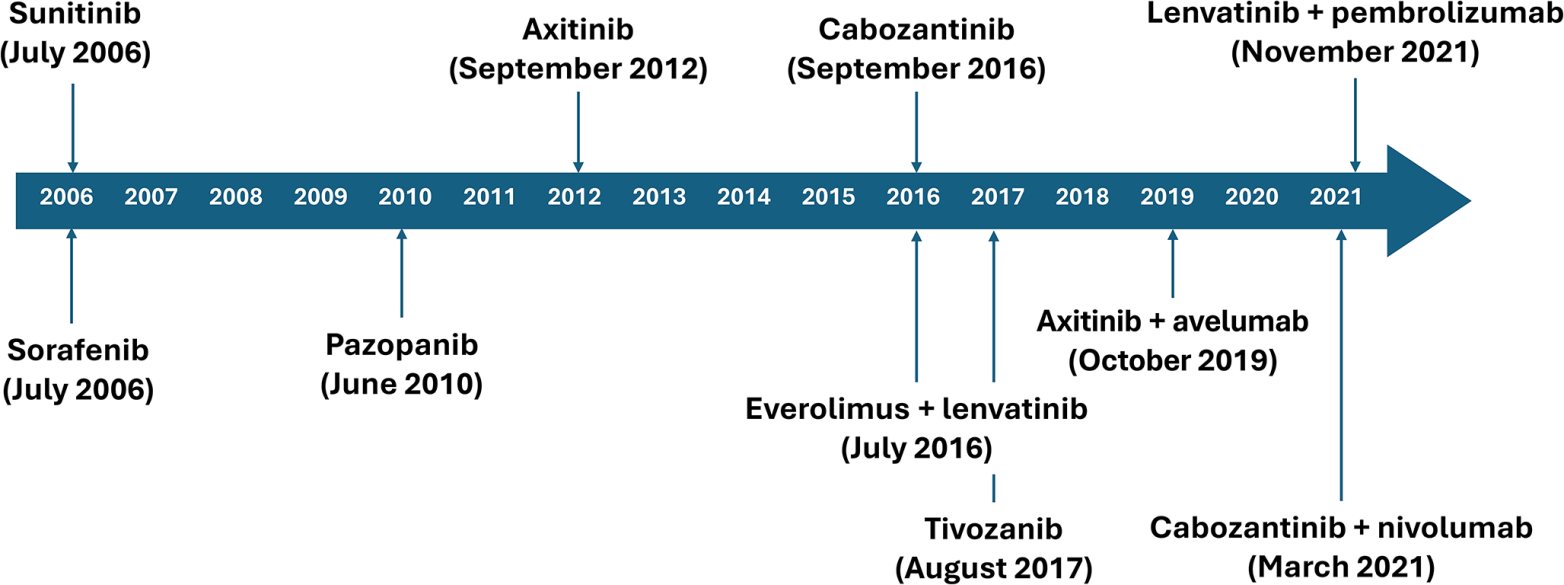
Timeline of approval of TKIs in Europe [56, 63, 74–81]

The treatment landscape for mRCC has changed substantially over the past 20 years with advances in the understanding of the biological and molecular basis of RCC leading to the development and approval of new targeted agents [8]. Starting in 2005, the approval of TKIs, such as sorafenib and sunitinib, heralded the availability of targeted treatment for mRCC, leading to TKIs becoming the gold standard of treatment [9] (Fig. 1). A major target of TKIs is vascular endothelial growth factor receptor (VEGFR) kinase, which plays a key role in the development and progression of RCC and a large number of other solid tumors. As an angiogenic protein, VEGF stimulates the growth of new blood vessels in tumors and the inhibition of its receptors (VEGFR-1, -2, and -3) thus blocks angiogenesis and tumor progression [10]. The availability of targeted therapies for mRCC was further expanded with the introduction of inhibitors of the mammalian target of rapamycin (mTOR) pathway, such as temsirolimus and everolimus [1].

The latest additions to the therapeutic landscape for mRCC are CPIs, specifically inhibitors of programmed death protein 1 (PD-1), programmed death-ligand 1 (PD-L1), and cytotoxic T-lymphocyte-associated antigen 4 (CTLA-4) [11]. By improving immune responses against cancer cells, CPIs such as nivolumab (PD-1 inhibitor) and ipilimumab (CTLA-4 inhibitor) have notably expanded the treatment options for patients with mRCC [7]. Since 2018, combination therapy with TKI/CPI and CPI/CPI regimens have become the standard of care for mRCC in the frontline setting. For example, four TKI/CPI combinations (axitinib plus avelumab; axitinib plus pembrolizumab; cabozantinib plus nivolumab; and lenvatinib plus pembrolizumab) and one CPI/CPI combination (ipilimumab plus nivolumab) are approved for mRCC by the US Food and Drug Administration (FDA) [12, 13] and the European Medicines Agency (EMA) [14].

New treatment options continue to emerge for mRCC, such as the hypoxia-inducible factor 2α (HIF-2α) inhibitor belzutifan, which was approved by the FDA in December 2023 for patients with mRCC whose disease had progressed following CPI and TKI treatment [15] and is currently under evaluation by the EMA [16]. However, even with the advent of belzutifan, which has a different mechanism of action to TKIs, targeting angiogenesis with TKIs remains a successful therapeutic target for mRCC. Furthermore, TKIs remain the backbone of treatment for mRCC, either in combination with CPIs or as monotherapy, in the first- and later-line settings.

## Making Treatment Decisions in mRCC

Patients with mRCC face complex treatment decisions influenced by various factors, including age, comorbidities, and concomitant medications; as well as individual preferences related to issues such as route of administration, types and rates of different adverse events (AEs), managing side effects, quality of life, and efficacy outcomes.

### Age Considerations

Aggressive therapeutic approaches may not be appropriate in all chronological or physiologically older patients due to toxicity and life expectancy [17]. Older patients may prefer to remain independent and receive oral medication rather than requiring regular hospital visits to receive infusions. In these patients, maintaining quality of life is often prioritized. Analysis of real-world first-line treatment choices for patients with mRCC suggests that physicians tend to emphasize treatment efficacy to patients above potential toxicity and quality of life [18].

Patient age at first-line treatment is important when considering the efficacy of a CPI-containing regimen or TKI monotherapy: in a meta-analysis of five studies comparing first-line CPI-containing regimens to sunitinib alone, younger patients (< 65 years) had significantly longer overall survival (OS) than patients aged ≥ 65 years (*p* = 0.007) and patients aged ≥ 75 years did not have any improvement in OS with CPI-based treatment [19].

### mRCC Risk Status

Determining a treatment plan for patients with mRCC starts with defining their risk status. Risk is stratified as favorable, intermediate, or poor and is defined by two models, the Memorial Sloan Kettering Cancer Center (MSKCC) model and the IMDC model [20–22]. The MSKCC model evaluates risk based on time from diagnosis to systemic treatment, Karnofsky performance status, and levels of hemoglobin, calcium, and lactate dehydrogenase (LDH) [20]. The more recent IMDC model, which is now in routine practice and thus will be focused on here, calculates risk based on time from diagnosis to systemic treatment, Karnofsky performance status, and levels of hemoglobin, calcium, platelets, and neutrophils [20–22]. Although important in risk stratification and treatment decisions, the MSKCC and IMDC models were developed before the emergence of CPIs into standard care and may need updating to include additional variables that may impact response to the evolving therapeutic landscape.

### Treatment Sequence

One of the concerns of starting treatment with TKI monotherapy in the first-line setting is the possibility of patients not receiving second-line therapy on progression, particularly if primary progression is seen. However, primary progression rates are less than 20% with single TKI, similar to combination CPI treatment, and real-world data sets such as UK Renal Oncology Collaborative [7] have demonstrated that drop-off rates between each line of therapy are lower in favorable risk than in intermediate and poor risk disease. Thus, in the favorable risk group, more patients are likely to be able to receive immunotherapy in the second-line setting compared with intermediate and poor risk patients, even if they progress through first-line treatment.

### Angiogenic/Immunogenic Signatures

In addition to assessing the patients’ risk status, the presence of angiogenic or immunogenic gene signatures may also help identify the most suitable treatment for individual patients, although this consideration has not yet entered routine clinical practice. As mentioned above, angiogenesis plays an important role in the development of RCC, which was highlighted by the discovery of the von Hippel-Lindau (*VHL*) gene. The *VHL* gene is dysfunctional in approximately 80% of cases of clear cell RCC [23, 24] and when the *VHL* gene is inactivated, HIF is not degraded in the proteasome and accumulates, resulting in the activation of the downstream signaling pathways influencing angiogenesis, cellular metabolism, and proliferation [25–27]. Immunological factors are also involved in tumor progression and prognosis, especially in clear cell RCC, which is an immunogenic tumor with a large number of infiltrating immune cells [28].

Tumors associated with favorable risk parameters are more likely to be angiogenically-driven and respond better to TKI therapy, while those categorized as poor risk tend to be enriched for an inflamed, more immune-driven phenotype with increased benefit from immunotherapy [29–32]. More recently, in a study on the molecular subtypes of 823 tumors from patients with mRCC treated with atezolizumab plus bevacizumab or sunitinib, seven distinct molecular clusters were identified (1. angiogenic/stromal; 2. angiogenic; 3. complement cascade/Ω-oxidation; 4. T-effector/proliferative; 5. proliferative; 6. stromal/proliferative; 7. small nucleolar ribonucleic acid) [33]. These molecular clusters were evaluated for their association with MSKCC and IMDC risk categories; as expected, in the favorable risk groups there was an enrichment of angiogenic/stromal (cluster 1) and angiogenic/fatty acid oxidation (cluster 2) molecular clusters, while in the poor risk groups, the enriched clusters were the T-effector/proliferative (cluster 4), proliferative (cluster 5), and stromal/proliferative (cluster 6) molecular subtypes [33]. Patients in both atezolizumab plus bevacizumab and sunitinib arms with angiogenesis-enriched clusters 1 and 2 had superior prognoses and similar progression-free survival (PFS), most likely due to the presence of an angiogenesis inhibitor in both arms. Conversely, atezolizumab plus bevacizumab significantly improved objective response rate (ORR) and PFS compared with sunitinib in patients with tumors exhibiting molecular clusters 4 and 5, consistent with the CPI-containing combination regimen for these angiogenesis-poor/immune-rich clusters [33, 34].

Additional evidence supporting the use of different gene signatures to further stratify different risk groups was provided by recent trials on predictive biomarkers. For example, the Phase 3 trial CheckMate 214 evaluated frontline ipilimumab plus nivolumab compared with sunitinib in patients stratified by IMDC risk groups [35]. Whereas ipilimumab plus nivolumab was efficacious across all risk groups and superior over sunitinib in patients with intermediate or poor risk, sunitinib demonstrated favorable outcomes in good risk patients [35]. In addition, the open-label, non-comparative, randomized, Phase 2 BIONIKK trial evaluated nivolumab, nivolumab plus ipilimumab, and sunitinib or pazopanib as frontline treatment for mRCC and aimed to show the feasibility of prospective patient and treatment selection based on tumor gene expression [36, 37]. Patients were divided into four molecular groups which were primarily characterized by distinct responses to sunitinib or pazopanib as well as distinct immune cell compositions and inhibitory receptor expressions based on a 35-gene expression signature [36, 37]. The BIONIKK trial is evaluating the personalization of mRCC treatment with TKI or CPI according to the molecular characteristics of the tumor and helps to identify biomarkers of response to nivolumab used alone or in combination, or to TKI monotherapy [36, 37]. Similarly, the OPTIC RCC trial is using the gene expression clusters to assign patients with mRCC to treatment with either a CPI/CPI combination regimen or a TKI/CPI combination treatment [38].

There is also increasing data to suggest that molecular signatures may be more important than PD-L1 status in mRCC. For example, in a biomarker analysis in the Phase 3 JAVELIN Renal 101 study, tumor samples were characterized by CD8 expression, gene expression signatures (26-gene JAVELIN Renal 101 signature), mutations, and polymorphisms [39]. Results demonstrated that patients with high-angiogenesis gene expression signature had significantly longer PFS in the sunitinib arm compared with the avelumab plus axitinib arm, while in the low-angiogenesis gene expression signature subset, PFS was longer in the avelumab plus axitinib arm [39]. Furthermore, higher numbers of CD8-positive cells were associated with longer PFS in the avelumab plus axitinib arm and with shorter PFS in the sunitinib arm [39]. In addition, an elevated expression of the immune gene expression signature correlated with greater PFS in the avelumab plus axitinib combination arm [39]. Thus, the absence of immune gene expression signatures could be an argument not to intensify treatment beyond TKI monotherapy.

Additional evidence is provided by the Phase 3 IMmotion151 trial that evaluated angiogenesis and T-effector gene expression signatures in patients treated with atezolizumab alone or combined with bevacizumab compared with sunitinib in the frontline setting [29]. The combination of atezolizumab plus bevacizumab improved PFS versus sunitinib in tumors that were T-effector cell high and low angiogenesis gene signature tumors [29]. In contrast, sunitinib improved PFS in patients with high angiogenesis gene signature tumors versus low angiogenesis gene signature tumors [29]. Furthermore, the favorable risk group could be characterized by a predominantly high angiogenesis gene signature [29].

Based on the findings of these studies, TKIs might be especially suitable for patients with favorable risk/angiogenic signatures, and CPIs for those with poor risk/immunogenic signatures.

## mRCC Treatment Options in the Frontline Setting

### TKI Monotherapy

The single-agent TKIs are recommended as valid options and should be discussed as stated in the guidelines by the European Society for Medical Oncology (ESMO) [3], the European Association of Urology (EAU) [2], the National Comprehensive Cancer Network (NCCN) [4] and the German guidelines [5] for mRCC in the first-line setting, including cabozantinib (EAU, NCCN, German guidelines), pazopanib (ESMO, EAU, NCCN, German guidelines), sunitinib (ESMO, EAU, NCCN, German guidelines), and tivozanib (ESMO, German guidelines) (Table 1) [2–4]. In addition, cabozantinib, lenvatinib, and axitinib can be used as part of TKI/CPI doublet therapies (Fig. 1) [3, 40]. Tivozanib is a potent and selective inhibitor of VEGFR-1, -2 and -3, whilst cabozantinib, pazopanib, and sunitinib target several additional tyrosine kinases, such as c-KIT receptor, platelet-derived growth factor receptor and FLT3 receptor [40–42].

**Table 1:** Response and survival outcomes for the single-agent TKIs recommended by ESMO [3], EAU [2], NCCN [4], and the German guidelines [5] in the frontline mRCC setting. Data in the table are based on the pivotal studies for cabozantinib [48, 49], pazopanib [43–45, 50, 72], sunitinib [46, 47, 51], and tivozanib [52, 53, 73]

	Cabozantinib	Pazopanib	Sunitinib	Tivozanib
**Median PFS, months (95% CI)**				
ITT population	8.6 (6.8–14.0)	9.2 (7.4–12.9)	11.0 (10.0–12.0)	11.9 (9.3–14.7)
MSKCC risk group				
Favorable	n/a	14.8 (*n*= 113 [39%]	n/a	16.7 (*n*= 70 [27%])
Intermediate	n/a	5.6 (*n*= 159 [54.8%])	n/a	9.4 (*n*= 173 [67%])
Poor	n/a	Not reported	n/a	3.7 (*n*= 17 [7%])
IMDC risk group				
Favorable	Excluded^a^	n/a	14.0 (*n*= 134 [37.5%])	NE (16.7–NR) (*n*= 41 [16%])
Intermediate	11.4 (*n*= 64 [81.0%])	n/a	10.7 (*n*= 205 [54.7%])	13.0 (*n*= 137 [53%])
Poor	6.8 (*n*= 15 [19%])	n/a	2.4 (*n*= 34 [11%])	Excluded^b^ (*n*= 78 [30%])
**Median OS, months (95% CI)**				
ITT population	26.6 (14.6–NE)	22.9	26.4 (23.0–32.9)	28.8 (22.5–NE)
**Response rate (%)**				
Overall response rate	20	30	47	33
Complete response	0	< 1	3	1
Partial response	20	30	44	32
Stable disease	54	38	40	52
Disease control rate	74	68	87	85

CI, confidence interval; EAU, European Association of Urology; ESMO, European Society for Medical Oncology; IMDC, International Metastatic RCC Database Consortium; ITT, intention-to-treat; MSKCC, Memorial Sloan Kettering Cancer Center; mRCC, metastatic renal cell carcinoma; n/a, not available; NCCN, National Comprehensive Cancer Network; NE, not estimable; NR, not reached; OS, overall survival; PFS, progression-free survival

^a^The CABOSUN trial included patients with intermediate or poor risk but not favorable risk mRCC. ^b^Sample size too small

When used as monotherapies in the frontline setting, pazopanib, sunitinib, and tivozanib have shown comparable anti-tumor efficacy in clinical trials (Table 1), despite their differing potency and selectivity for VEGFR [3, 40]. Rates of disease control (patients achieving stable disease or better) across the TKIs approved as first-line monotherapies were high (68–87%; Table 1) [43–53]. With tivozanib, disease control was achieved by 85% of patients receiving frontline monotherapy, while only 13% of patients experienced progression as the primary response [52]. The selectivity of tivozanib [40–42] compared with the other first-line TKIs may account for lower rates of off-target side effects such as diarrhea, fatigue, and hand-foot syndrome (Table 2). Thus, for those patients in whom a single-agent TKI is suitable, tivozanib allows good control of disease and demonstrates a favorable toxicity profile compared with other single agent TKIs and combination therapies.

### TKI Monotherapy Versus Combination Therapies

TKI monotherapy is likely to be more effective in tumors that are angiogenesis-driven, while regimens containing CPI are more likely to be effective in tumors that are more immune-driven. In clinical trials comparing mRCC therapies, particularly the trials of combination therapies, sunitinib is used as the active comparator and performs similarly across different trials [35–37].

Independent of the drug used, efficacy of TKI monotherapy has been observed as being consistent across IMDC favorable risk groups, while TKI/CPI and CPI/CPI combination regimens may be in particular beneficial in patients with intermediate and poor risk disease, because most patients included in the pivotal trials belonged to these risk categories. For example, in the CheckMate 214 study, patients with intermediate or poor risk demonstrated significantly higher OS and ORR in the nivolumab plus ipilimumab group than in the sunitinib group [35]. In the favorable risk group, frontline combination therapy resulted in modest benefits with respect to PFS and ORR, in comparison to TKI monotherapy. OS benefits are not clear in this risk group [35]. Despite the fact that long-term follow-up of the pivotal trial after at least 5-years revealed additional events in the favorable risk population, the data remain elusive [54]. Furthermore, significant focus is often placed upon the importance of increased complete response rate; however, in the JAVELIN Renal 101 trial the difference in complete response rate was less than 2% with the absolute numbers of 4.8% in the TKI/CPI arm compared with 3.2% in single agent TKI arm [55]. In the final analysis of OS after > 5 years follow up in JAVELIN Renal 101, median OS was 44.8 months (95% confidence interval [CI] 39.7–51.1) for avelumab with axitinib and 38.9 months (95% CI 31.4–45.2) for sunitinib alone (hazard ratio [HR] 0.88, 95% CI 0.749–1.039; *p* = 0.067) [56]. Moreover, most complete responses will ultimately progress and thus the palliative nature of systemic anti-cancer therapies in treatment of metastatic disease should not be forgotten when discussing efficacy end points with patients. As outlined previously, many patients in real-world clinical practice prioritize OS as the goal of treatment and it is also considered the gold standard measure in most settings by physicians. However, if OS is not improved with TKI/CPI combination therapy in the favorable risk groups, then sequencing treatment with frontline TKI monotherapy to reduce the increased toxicity of TKI/CPI combination treatments in the frontline may be a better treatment approach, particularly for those patients who are prioritizing medication with lower toxicity. Indeed, in the real-world CARAT registry study, OS benefits were not observed in the TKI/CPI combination group compared with single-agent TKI treatment in an inverse probability of treatment weighting adjusted analysis [57].

In a network meta-analysis of four RCTs, TKI/CPI combinations for frontline mRCC treatment improved PFS but not OS compared with sunitinib in patients with favorable IMDC prognosis [58], which has led to a position of uncertainty in the renal community about the preferred treatment option for favorable risk patients. TKI/CPI combinations resulted in better ORR than sunitinib (60.4% vs. 39.6% for pembrolizumab plus axitinib vs. sunitinib; 54.8% vs. 28.4% for nivolumab plus cabozantinib vs. sunitinib; 67.0% vs. 39.6% for avelumab plus axitinib vs. sunitinib; and 71.0% vs. 36.1% for pembrolizumab plus lenvatinib vs. sunitinib) [58]. TKI/CPI combinations improved PFS compared with sunitinib (HR 0.63; *p* < 0.00001); however, OS was not significantly prolonged (HR 0.99; 95% CI 0.74–1.33; *p* = 0.95) [58].

In an updated report of this meta-analysis with longer follow-up, results confirmed that the TKI/CPI combination regimens did not significantly improve OS in mRCC, while improvements in PFS remained significant [59]. Thus, when making treatment decisions in patients with favorable mRCC prognosis, the longer expected PFS and greater risk of toxicity with CPI combination therapies, without added OS benefit, should be considered [59].

An FDA pooled analysis of first-line TKI/CPI combination therapy versus sunitinib in mRCC by IMDC risk group also concluded that OS benefit is yet to be demonstrated in the favorable risk group (HR 1.24; 95% CI 0.86–1.78) and the HR has tended to worsen over time [12]. However, an OS benefit was seen in the pooled intermediate/poor risk group (HR 0.64; 95% CI 0.55–0.75) [12]. For PFS, there was a benefit for TKI/CPI in the favorable risk group (HR 0.63; 95% CI 0.50–0.79) and the intermediate/poor risk group (HR 0.52; 95% CI 0.45–0.60) [12]. A smaller difference was observed in ORR between the TKI/CPI and sunitinib arms in the favorable risk group (68.2% vs. 49.9%) compared with the intermediate/poor risk group (59.9% vs. 36.5%), while the difference in complete response was larger for favorable risk (15.3% vs. 6.0%) than for intermediate/poor risk patients (9.1% vs. 3.4%) [12].

The more complex safety profile associated with combination therapy further favors the use of TKI monotherapy in the first-line setting. CPIs exhibit distinct AE profiles compared with TKIs. The most common AEs associated with CPIs are immune-related AEs, such as rash, pruritus, and pneumonitis, while common AEs with TKIs include diarrhea, hypertension, and hand-foot syndrome [60]. While TKI toxicity typically resolves on stopping treatment, there are a number of life-threatening and permanent toxicities that can occur in patients who receive immunotherapy [61, 62].

## Tivozanib Monotherapy for mRCC in the Frontline Setting

Tivozanib is approved by the EMA for the treatment of adults with mRCC in the frontline setting and for second-line treatment of patients with mRCC who are VEGFR and mTOR pathway inhibitor-naïve following disease progression after one prior treatment with cytokine therapy [63]. In the US, tivozanib is approved for adult patients with relapsed or refractory mRCC after two or more prior systemic therapies [64].

Tivozanib has similar efficacy compared with other TKIs in the frontline setting (Table 1); however, its more favorable safety profile (Table 2) [40] and thus the better daily life of patients receiving this therapy may be a key differentiator when making treatment decisions related to TKI monotherapy.

**Table 2:** Tolerability profiles for the single-agent TKIs recommended by ESMO [3], EAU [2], NCCN [4], and the German guidelines [5] in the frontline mRCC setting. Data in table based on the pivotal studies for cabozantinib [48, 49], pazopanib [44], sunitinib [46, 47], and tivozanib [52]

	Cabozantinib	Pazopanib	Sunitinib	Tivozanib
**Dose reductions (%)**	46	NR	38	14
**Dose interruptions (%)**	NR	NR	32	19
**Discontinuation due to AE (%)**	20	14	8	4
**Off-target AEs (%)**				
Fatigue				
All grades	86	19	54	19
Grades 3/4	6	2	11	5
Hand-foot syndrome				
All grades	42	NR	29	14
Grades 3/4	8	NR	9	2
Diarrhea				
All grades	72	52	61	23
Grades 3/4	10	4	9	2

AE, adverse event; EAU, European Association of Urology; ESMO, European Society for Medical Oncology; mRCC, metastatic renal cell carcinoma; NCCN, National Comprehensive Cancer Network; NR, not reported

### Tivozanib Clinical Efficacy

The efficacy and safety of tivozanib were evaluated in the Phase 3 trial TIVO-1, which compared tivozanib monotherapy with sorafenib (active comparator) as frontline treatment in patients with mRCC (Table 1) [52]. Median PFS was longer in patients receiving tivozanib (*n* = 260) than in patients receiving sorafenib (*n* = 257; 11.9 vs. 9.1 months, respectively; HR 0.80; 95% CI 0.64–0.99; *p* = 0.042) [52]. In a subgroup analysis by MSKCC risk score, there was an advantage with tivozanib treatment in patients with favorable or intermediate risk; the ORR for tivozanib was 33.1% (95% CI 27.4–39.2%) compared with 23.3% (95% CI 18.3–29.0%) for sorafenib (*p* = 0.014), and there was a trend for longer median OS in the sorafenib group compared with the tivozanib group (29.3 vs. 28.8 months; HR 1.25; 95% CI 0.95–1.62; *p* = 0.105) [52]. An exploratory subgroup analysis of the TIVO-1 trial found significant improvement in median PFS with tivozanib versus sorafenib in the IMDC favorable (HR 0.39, *p* = 0.003) and intermediate prognostic groups (HR 0.74, *p* = 0.044) [53].

In a *post-hoc* analysis, which excluded the patients enrolled in eastern Europe due to the lower proportion of patients in those regions receiving second-line targeted therapy as part of standard of care, median OS was 32.9 months for tivozanib compared with 29.5 months for sorafenib in the 186 patients enrolled in North America and Europe (HR 0.85; *p* = 0.433) [65]. The proportion of patients receiving second-line targeted treatment was more balanced in the *post-hoc* analysis populations (55.6% vs. 79.5% for tivozanib and sorafenib, respectively) than in the intention-to-treat population (38.4% vs. 75.7% for tivozanib and sorafenib, respectively) [65].

### Tivozanib Safety and Tolerability

Tivozanib is a more potent and selective VEGFR TKI than sorafenib, with a longer half-life [66, 67], and in the TIVO-1 trial, tivozanib demonstrated a more differentiated safety profile compared with sorafenib [52]. Due to its selective mechanism of action, off-target side effects, such as diarrhea, fatigue, and hand-foot syndrome, are lower compared with multi-kinase TKIs (Table 2).

Furthermore, in the meta-analysis of TKIs discussed earlier, it was not possible to produce a clear hierarchy of frontline TKIs based on efficacy, and the authors of that analysis suggested that toxicity may play a more significant role in treatment decisions [40]. The analysis indicated that tivozanib had the most favorable safety profile and was associated with significantly less risk of toxicity than other TKIs [40], which is consistent with the high specificity for VEGFR of tivozanib compared with other TKIs leading to fewer off-target side effects [41, 42]. Similarly, in a network meta-analysis comparing the efficacy and safety of approved frontline TKIs for mRCC in 12 studies, tivozanib was associated with a more favorable safety profile (fewer grade 3 or 4 toxicities) than cabozantinib, sunitinib, and pazopanib, while there were no significant differences between TKIs in efficacy [40]. Additionally, rates of discontinuation due to AEs are low at 4% in patients treated with tivozanib [52], which is higher than the discontinuation rate seen with other TKIs (Table 2) and is supportive of tivozanib’s tolerability.

### Tivozanib Quality of Life

In the TIVO-1 trial of tivozanib versus sorafenib, health-related quality of life was maintained throughout the first 12 months of treatment in both TKI treatment groups [52]. Additionally, the improved tolerability of tivozanib was associated with improved patient quality of life through fewer side effects and simplified dosing with fewer dose interruptions or dose reductions for AE management [42].

Furthermore, the AEs most frequently reported in clinical trials may not match the side effects that most negatively impact quality of life. For example, in the T-REX real-world study of patients with mRCC treated with tivozanib in the frontline setting in Germany, the most common (> 10%) physician-reported AEs in patients were diarrhea, nausea, and hypo- or hypertension [39, 40]. In contrast, the most common patient-reported AEs that interfered with usual activities or daily life were fatigue, shortness of breath, and problems with concentration [39, 40]. This disconnect between the physician-reported AEs and patient-reported side effects affecting usual activities was also evident even for AEs graded 3/4 in severity. For example, the most common physician-reported grade 3/4 AEs were diarrhea and cardiac dysfunction, while patients reported fatigue and dry mouth most often as grade 3/4 side effects [39, 40].

### Tivozanib Real-World Evidence

The disconnect between the AEs that are reported by physicians and those that matter most to patients in their everyday lives highlights the importance of real-world data. Moreover, analyses from the German Clinical RCC Registry has found that as many as 57% of patients are not eligible for clinical trials [68], so evaluating response, outcomes, and tolerability in the full patient population in real-world clinical settings is key.

Real-world data confirmed the efficacy and tolerability profile of tivozanib monotherapy in the frontline setting in various countries. A retrospective, real-world analysis was conducted that evaluated 64 patients with mRCC receiving tivozanib in a compassionate use program in Italy [69]. In this study, 34.4% of patients responded and 40.6% of patients had stable disease, with a median PFS of 12.4 months and 68.7% of patients alive at 12 months [69]. In a retrospective, real-world study in the UK, 113 patients with mRCC were treated with frontline tivozanib, 26% of whom were switched to tivozanib from other TKIs due to toxicity; data were comparable to those of the pivotal trial [7]. After a median follow-up of 26.6 months, median PFS was 8.75 months and median OS was 25.0 months. When stratified by IMDC risk group, median PFS was 23.0 months, 10.0 months, and 3.0 months in the favorable, intermediate, and poor risk groups, respectively. Median OS was not reached in the IMDC favorable risk group, with 72% alive at data cut-off, and was 26.0 months in the intermediate risk group and 7.0 months in the poor risk group [7]. AEs of any grade were experienced by 77% of patients and grade ≥ 3 events by 13% of patients [7]. A further retrospective real-world study of patients with mRCC treated with frontline tivozanib in Spain reported higher PFS (21 months) and OS (30 months) than the TIVO-1 trial, with similar ORR and safety profile [70]. In addition, in the prospective, non-interventional T-REX study of tivozanib in patients with mRCC across real-world clinical practices in Germany (*N* = 32), first-line treatment with tivozanib resulted in an ORR of 46.9%, with a generally favorable tolerability profile. Furthermore, the German CARAT registry study [71] showed a median PFS of 20.0 months (95% CI 3.2–not estimable); median OS was not reached at time of the analysis of 24 patients (median age 77.6 years) receiving frontline tivozanib in routine care (unpublished data; personal communication, N. Marschner).

From clinical trial data and real-world studies, it can be seen that similar response rates, PFS, and OS are observed across risk groups and different TKI options in the frontline setting. If efficacy is comparable, then treatment choices between TKIs should consider tolerability and quality of life. As discussed previously, tivozanib is a potent and selective VEGFR inhibitor, which may provide advantages over less-selective TKIs that bind to other non-VEGF dependent receptors.

## Conclusions

In patients with mRCC, first-line treatment with CPI/CPI and TKI/CPI combinations in patients with intermediate or poor IMDC prognosis or with TKI/CPI combinations in the overall population has been shown to increase OS compared with single-agent TKI treatment and is, therefore, considered standard of care in treatment guidelines for each of these risk groups.

However, the best treatment approach for patients with favorable risk remains uncertain because no clear difference in OS between CPI-containing combination therapies and TKI monotherapies have been identified. Patients with favorable risk at diagnosis tend to have biologically less aggressive, slower-growing, low-volume disease and therefore usually present a clinical situation that rarely requires an urgent response as part of the therapeutic strategy. In addition, single agent TKIs, in particular tivozanib, has the advantage of lower toxicity rates and most patients who progress will remain able to receive nivolumab or cabozantinib in the second-line setting.

Tivozanib has shown efficacy in the first-line treatment of patients with mRCC in both Phase 3 clinical trials and real-world evidence studies. Its advantages over other TKIs in terms of greater anti-angiogenic specificity, lower rate of side effects and fewer drug interactions make it an ideal candidate for the treatment of these patients.

As well as providing the backbone to the majority of combination therapies, TKIs also remain a viable treatment option as monotherapy in the frontline setting of patients with mRCC in the favorable risk group and those with contraindications to CPI combinations. In the absence of OS benefit of CPI combination regimens, even over the longer term [12], we argue that frontline TKI monotherapy, such as tivozanib, remains a standard of care option for favorable risk patients, which is supported by clinical treatment guidelines [2–4].

## Key References

2. Ljungberg B, Albiges L, Abu-Ghanem Y, Bedke J, et al. European Association of Urology guidelines on renal cell carcinoma: The 2022 update. Eur Urol. 2022;82(4):399–410.

- Of outstanding importance: The latest EAU guidelines and recommendations on the management of RCC. Includes the option of single-agent TKIs as first-line treatment for mRCC.

3. Powles T, Albiges L, Bex A, Comperat E, et al. Renal cell carcinoma: ESMO Clinical Practice Guideline for diagnosis, treatment and follow-up. Ann Oncol. 2024;S0923-7534(24)00676-8.

- Of outstanding importance: The current clinical practice guideline for the diagnosis, treatment and follow-up of RCC from ESMO. States that treatment options in the first-line setting for mRCC include single-agent TKIs, such as tivozanib.

4. Motzer RJ, Jonasch E, Agarwal N, Alva A, et al. NCCN Guidelines^®^ insights: Kidney cancer, version 2.2024. J Natl Compr Canc Netw. 2024;22(1):4–16.

- Of outstanding importance: The latest recommendations for diagnostic workup, staging, and treatment of patients with RCC from the NCCN. Confirms that single-agent TKIs are a first-line treatment option for mRCC.

7. Heseltine J, Allison J, Wong S, Prasad K, et al. Clinical Outcomes of Tivozanib Monotherapy as First-Line Treatment for Metastatic Renal Cell Carcinoma: A Multicentric UK Real-World Analysis. Target Oncol. 2023;18(4):593-9.

- Of importance: A recent real-world study of 113 patients with mRCC receiving tivozanib monotherapy in the first-line setting. Results demonstrate that tivozanib is an effective first-line treatment option for mRCC in clinical practice and that its tolerability profile suggests that it is an appropriate treatment option for patients who are not suitable for combination therapies or other TKIs.

20. Aldin A, Besiroglu B, Adams A, Monsef I, et al. First-line therapy for adults with advanced renal cell carcinoma: a systematic review and network meta-analysis. Cochrane Database Syst Rev. 2023;5(5):Cd013798.

- Of importance: A Cochrane systematic literature review and network meta-analysis of 36 randomized controlled trials (15,177 participants) of first-line treatments for advanced RCC. Trial evaluating at least one targeted therapy or immunotherapy (including combination therapies) were included. Results highlight the importance of assessing outcomes by risk groups.

## Data Availability

No datasets were generated or analysed during the current study.
