# Correction to: Tivozanib Monotherapy in the Frontline Setting for Patients with Metastatic Renal Cell Carcinoma and Favorable Prognosis

**DOI:** 10.1007/s11912-025-01670-6

**Published:** 2025-05-09

**Authors:** Ricky Frazer, José Ángel Arranz, Sergio Vázquez Estévez, Omi Parikh, Laura-Maria Krabbe, Naveen S. Vasudev, Christian Doehn, Norbert Marschner, Tom Waddell, Will Ince, Peter J. Goebell

**Affiliations:** 1 Velindre Cancer Centre, Cardiff, CF14 2TL UK; 2 Hospital General Universitario Gregorio Marañón, Madrid, Spain; 3 Hospital Universitario Lucus Augusti (HULA), Lugo, Spain; 4 Lancashire Teaching Hospitals NHS Foundation Trust, Chorley, UK; 5 Klinik für Urologie, Vivantes Humboldt-Klinikum, Berlin, Germany; 6 Leeds Institute of Medical Research at St James’S, Leeds, UK; 7 Urologikum Lübeck, Lübeck, Germany; 8 Clinical Research Institute IOMEDICO, Freiburg, Germany; 9 The Christie NHS Foundation Trust, Manchester, UK; 10 Department of Oncology, Cambridge University NHS Foundation Trust, Cambridge, UK; 11 Uniklinik Erlangen, Urologische und Kinderurologische Klinik, Erlangen, Germany

**Correction to: Current Oncology Reports (2024) 26:1639–1650**

10.1007/s11912-024-01613-7

The original online version of this article was revised due to formatting errors introduced by the journal during typesetting.

1. Updates to the tables regarding bold emphasis and alignment have been made as shown below.

**Table 1:** Response and survival outcomes for the single-agent TKIs recommended by the European Society for Medical Oncology [3], the European Association of Urology [2], the National Comprehensive Cancer Network [4] and the German guidelines [5] in the frontline mRCC setting. Data in the table are based on the pivotal studies for cabozantinib [48, 49], pazopanib [43–45, 50, 72], sunitinib [46, 47, 51], and tivozanib [52, 53, 73]

	Cabozantinib	Pazopanib	Sunitinib	Tivozanib
**Median PFS, months (95% CI)**				
ITT population	8.6 (6.8–14.0)	9.2 (7.4–12.9)	11.0 (10.0–12.0)	11.9 (9.3–14.7)
MSKCC risk group				
Favorable	n/a	14.8 (*n*=113 [39%]	n/a	16.7 (*n*=70 [27%])
Intermediate	n/a	5.6 (*n*=159 [54.8%])	n/a	9.4 (*n*=173 [67%])
Poor	n/a	Not reported	n/a	3.7 (*n*=17 [7%])
IMDC risk group				
Favorable	Excluded^a^	n/a	14.1 (*n*=134 [37.5%])	NE (16.7–NR) (*n*=41 [16%])
Intermediate	11.4 (*n*=64 [81.0%])	n/a	10.7 (*n*=205 [54.7%])	13.0 (*n*=137 [53%])
Poor	6.8 (*n*=15 [19%])	n/a	2.4 (*n*=34 [11%])	Excluded^b^ (*n*=78 [30%])
**Median OS, months (95% CI)**				
ITT population	26.6 (14.6–NE)	22.9	26.4 (23.0–32.9)	28.8 (22.5–NE)
**Response rate (%)**				
Overall response rate	20	30	47	33
Complete response	0	<1	3	1
Partial response	20	30	44	32
Stable disease	54	38	40	52
Disease control rate	74	68	87	85

*CI* confidence interval, *EAU* European Association of Urology, *ESMO* European Society for Medical Oncology, *IMDC* International Metastatic RCC Database Consortium, *ITT* intention-to-treat, *MSKCC* Memorial Sloan Kettering Cancer Center, *mRCC* metastatic renal cell carcinoma, *n*/*a* not available, *NCCN* National Comprehensive Cancer Network, *NE* not estimable, *NR* not reached, *OS* overall survival, *PFS* progression-free survival

^a^The CABOSUN trial included patients with intermediate or poor risk but not favorable risk mRCC. ^b^Sample size too small

**Table 2:** Tolerability profiles for the single-agent TKIs recommended by the European Society for Medical Oncology [3], the European Association of Urology [2], the National Comprehensive Cancer Network [4] and the German guidelines [5] in the frontline mRCC setting. Data in table based on the pivotal studies for cabozantinib [48, 49], pazopanib [44], sunitinib [46, 47], and tivozanib [52]

	Cabozantinib	Pazopanib	Sunitinib	Tivozanib
Dose reductions (%)	46	NR	38	14
Dose interruptions (%)	NR	NR	32	19
Discontinuation due to AE (%)	20	14	8	4
Off-target AEs (%)				
Fatigue				
All grades	86	19	54	19
Grades 3/4	6	2	11	5
Hand-foot syndrome				
All grades	42	NR	29	14
Grades 3/4	8	NR	9	2
Diarrhea				
All grades	72	52	61	23
Grades 3/4	10	4	9	2

*AE* adverse event, *EAU* European Association of Urology, *ESMO *European Society for Medical Oncology, *mRCC *metastatic renal cell carcinoma, *NCCN *National Comprehensive Cancer Network, *NR* not reported

2. In the updated Figure 1 below, the spell-check markings (red underlines) have been removed.

**Figure:**
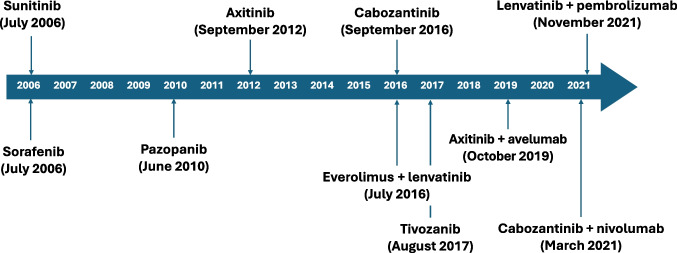

The original article has been corrected.

